# Gas Chromatography‐Mass Spectrometry Analysis, Genoprotective, and Antioxidant Potential of *Curio radicans* (L. f.) P.V. Heath

**DOI:** 10.1002/open.202500175

**Published:** 2025-06-08

**Authors:** Muhammad Naseer, Muhammad Adil, Sarir Ahmad, Mikhlid H. Almutairi, Abdulwahed Fahad Alrefaei, Sajid Ali, Fayaz Asad

**Affiliations:** ^1^ Department of Chemical and Life Sciences Qurtuba University of Science and Information Technology Peshawar Pakistan; ^2^ Department of Forestry University of Agriculture Dera Ismail Khan 29111 Pakistan; ^3^ College of Life and Environmental Sciences Central South University of Forestry and Technology Changsha 410004 China; ^4^ Zoology Department College of Science King Saud University P.O. Box: 2455 Riyadh 11451 Saudi Arabia; ^5^ Department of Zoology College of Science King Saud University P.O. Box 2455 Riyadh 2455 Saudi Arabia; ^6^ Department of Horticulture and Life Science Yeungnam University Gyeongsan 38541 Republic of Korea; ^7^ Department of Botany Bacha Khan University Charsadda 24420 Khyber Pakhtunkhwa Pakistan

**Keywords:** antioxidant activity, *Curio radicans*, gas chromatography‐mass spectrometry analysis, genotoxic

## Abstract

Natural substances play a crucial role in modern medicine by targeting cellular pathways associated with diseases. Medicinal plants, rich in bioactive compounds, offer promising therapeutic potential. This study examines the phytochemicals and biological activity of *Curio radicans* extracts. Gas chromatography‐mass spectrometry analysis identifies 29 compounds in the ethanolic extract and 7 in the ethyl acetate extract. The ethanolic extract contains 3,4‐Bis (Methoxycarbonyl)furan (15.76%) and 4‐Imidazolidinone (14.17%), while the ethyl acetate extract is dominated by Diisooctyl phthalate (49.25%). Genoprotective activity, using the comet assay on human lymphocytes, demonstrates significant dose‐dependent reductions in H_2_O_2_‐induced DNA damage. The ethyl acetate extract reduces total comet score (TCS) from 265.3 ± 20.4 to 33.0 ± 4.3 at 100 mg/100 mL (*p* < 0.0001), outperforming the ethanolic extract, which reduced TCS from 226.7 ± 41.6 to 44.0 ± 7.2 (*p* < 0.001). Antioxidant activity, assessed via the DPPH method, reveals moderate activity for the ethyl acetate extract (72.61 ± 1.65%, IC_50_: 137.58 μg/ml) and lower activity for the ethanolic extract (67.52 ± 0.44%, IC_50_: 156.52 μg/ml). These findings underscore the therapeutic potential of *C. radicans*, particularly the ethyl acetate extract, which demonstrates genoprotective effects. Researchers should focus on isolating active compounds and exploring their potential in drug development.

## Graphical abstract

The ethyl acetate extract of *Curio radicans* is found to contain a higher proportion of bioactive compounds (55% vs 30% in the ethanolic extract) by gas chromatography‐mass spectrometry, and it exhibits stronger antioxidant activity in the DPPH assay (75% vs 65% inhibition) as well as clear genoprotective effects against H_2_O_2_‐induced DNA damage, highlighting its potential as a source of natural protective agents.

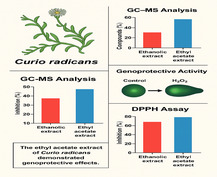

## 1 Introduction

Plants have long been recognized as valuable sources of medicinal compounds, with their primary and secondary metabolites responsible for various therapeutic properties. While primary metabolites are essential for the growth and development of plants, secondary metabolites play a crucial role in pharmacological activities, including antidiabetic, antibacterial, anti‐inflammatory, antiparasitic, antiviral, antitumor, and antinociceptive effects.^[^ ^1^ ^]^ Beyond medicinal applications, secondary metabolites also perform vital ecological functions such as defense against pathogens, attraction of pollinators and seed‐dispersing animals, UV protection, and allelopathic interactions. Furthermore, these compounds are used in the production of dyes, waxes, pharmaceuticals, perfumes, and other industrial products.^[^ ^2^ ^]^ Medicinal plants, with their diverse phytochemicals, act as invaluable reservoirs of bioactive molecules. These compounds not only exhibit therapeutic potential but also serve as precursors for the synthesis of novel drugs and pharmaceutical agents.^[^ ^3^ ^]^

Gas chromatography‐mass spectrometry (GC‐MS) is a powerful analytical technique that combines the separation capabilities of gas chromatography with the structural identification efficiency of mass spectrometry.^[^ ^4^ ^]^ This method is commonly used for the chemical analysis of plant extracts, enabling accurate identification of phytochemicals and aiding in the understanding of their pharmacological roles.^[^ ^5^ ^]^

Oxidation is a fundamental chemical process involving the transfer of electrons, which can become harmful when reactive oxygen species (ROS) accumulate excessively. This condition, known as oxidative stress, leads to significant damage to cells and tissues.^[^ ^6^ ^]^ Plant extracts rich in antioxidants have attracted considerable attention due to their low toxicity, wide availability, and therapeutic potential. Compounds such as polysaccharides, flavonoids, phenols, and pigments possess strong antioxidant properties, making them promising candidates for reducing oxidative stress and its associated health risks.^[^ ^7^ ^]^ One of the major consequences of oxidative stress is DNA damage, which, if left unrepaired, results in genomic instability, mutations, cancer, aging, and various other diseases. Chromosomal aberrations, sister chromatid exchanges, and micronucleus formation in human blood lymphocytes serve as key biomarkers for assessing genotoxic alterations and exposure to carcinogenic agents.^[^ ^8^ ^]^

*Curio radicans* (L. f.) P.V. Heath, commonly known as String of Bananas, Fish Hooks Senecio, or Fishhooks, belongs to the family Asteraceae. This popular trailing succulent typically flowers in late winter or early spring. It is native to Namibia, Lesotho, and South Africa, and has also been reported in the western regions of Pakistan, particularly in Balochistan. Adapted to arid and semi‐arid environments, *C. radicans* thrives in dry conditions and is often found growing along roadsides, slopes, and rocky habitats. Its ability to endure such challenging conditions reflects its ecological resilience. *C. radicans* exhibits medicinal potential, including antioxidant, antimicrobial, and cytotoxic properties. Its bioactive compounds contribute to disease prevention and support drug development. Traditionally, *C. radicans* has also been used for therapeutic purposes such as wound healing and reducing inflammation.^[^ ^9^ ^]^

This study aims to evaluate the pharmacological potential of *Curio radicans*, focusing on its phytochemical composition and antioxidant activity. By employing advanced analytical techniques such as GC‐MS, this research seeks to standardize the plant for medicinal use and explore its role in mitigating oxidative stress and genotoxic effects. The ultimate objective is to identify bioactive compounds that may serve as candidates for future drug development, contributing to the expanding field of plant‐based therapeutics.

## 2 Materials and Methods

### 2.1 Plant Collection

*Curio radicans* was collected in March 2024 from Quetta, Balochistan, Pakistan, located at 30.183270° N latitude and 66.996452° E longitude. The plant identification was confirmed using the *Flora of Pakistan* and with the assistance of Ghulam Jelani, a plant taxonomist. A specimen has been preserved in the herbarium for future reference under the voucher number Muhammad Naseer Bot.50 (QUSIT).

### 2.2 Plant Extraction

The selected plant was rinsed with distilled water, shade‐dried at room temperature, and ground into a fine powder. A 50 g sample of the powder was soaked in 250 mL each of ethanol and ethyl acetate solvents, supplied by U.M. Enterprises. After 48 h, the extract was strained through muslin cloth and then filtered using filter paper. The extract was concentrated using a rotary evaporator (RE‐100D Phoenix) from MED Lab Services. The resulting ethyl acetate extract (10.15 g) and ethanol extract (10.5 g) of *Curio radicans* were stored at 4 °C in sealed bottles for future use.^[^ ^10^ ^]^

### 2.3 GC‐MS

GC‐MS analysis of the *Curio radicans* crude extract was performed using a Thermo GC‐Trace Ultra version 5.0 coupled with a Thermo MSDSQII, both supplied by MED Lab Services. To prepare the sample, 2 mg of crude extract was dissolved in 5 mL of the respective solvents. The sample mixtures were purified using a ZB 5‐MS capillary nonpolar column (30 m, 0.25 mm ID, 0.25 μm film). The column temperature was initially set at 70 °C and increased by 2 °C per minute until reaching 260 °C, followed by a 10‐minute hold at 6 C per minute. The sample was injected in splitless mode (10 mL/min split flow, 1‐minute splitless time), with helium as the carrier gas at a constant flow rate of 1 mL/min, and 1 μL of the sample was injected. Peak area normalization was used to determine the relative percentages of the extract's components. The mass spectral scan range was set to 50–650 m/z in full scan mode. Compounds were identified by comparing their retention indices with reference compounds stored in the Wiley and Main Lab computer library search software.^[^ ^11^ ^]^

### 2.4 Genotoxic Activity

The genotoxic activity was assessed using the comet assay, following modifications of the method by Adil et al.^[^ ^8^ ^]^ Initially, the test substance is applied to cultured cells, which are then harvested and suspended in an appropriate buffer. The cells are embedded in a layer of low‐melting‐point agarose on a microscope slide, and electrophoresis is carried out to induce DNA migration after cell lysis to release biological components. The slide is then stained with a DNA‐specific fluorescent dye, and parameters such as tail length, tail intensity, and tail moment are analyzed using fluorescence microscopy and image analysis software. Genotoxicity is evaluated by comparing these measurements between the treatment groups and the control samples.

### 2.5 Antioxidant Activity

The antioxidant potential of *Curio radicans* ethanolic and ethyl acetate extracts was assessed using the DPPH assay. The methodology was adapted from ref. [12] with slight modifications. A 0.135 mM DPPH solution was prepared in methanol, and 1.0 mL of this solution was mixed with 1.0 mL of the plant extract at varying concentrations (100, 200, and 300 μg/mL). The reaction mixture was incubated at room temperature in the dark for 30 min to evaluate its capacity to inhibit ROS. Ascorbic acid was used as a reference standard. The control consisted of 1.0 mL of methanol mixed with 1.0 mL of DPPH solution. The assay was performed in triplicate, and absorbance reduction was measured at 517 nm using a UV–vis spectrophotometer at 30, 60, and 90 min. The percentage of inhibition was calculated using the following formula:

Inhibition % = Ac−As/Ac×100

where “Ac” is the absorbance of the control and “As” is the absorbance of the sample

### 2.6 Statistical Analysis

Data were analyzed using SPSS version 20. One‐way ANOVA was used to compare the groups following Tukey's test. Values were expressed as mean ± standard deviation (S.D.). The difference was considered significant relative to the positive control when *p < 0.01, **p < 0.002, ***p < 0.001.

## 3 Results

### 3.1 GC‐MS

**Figure 1:**
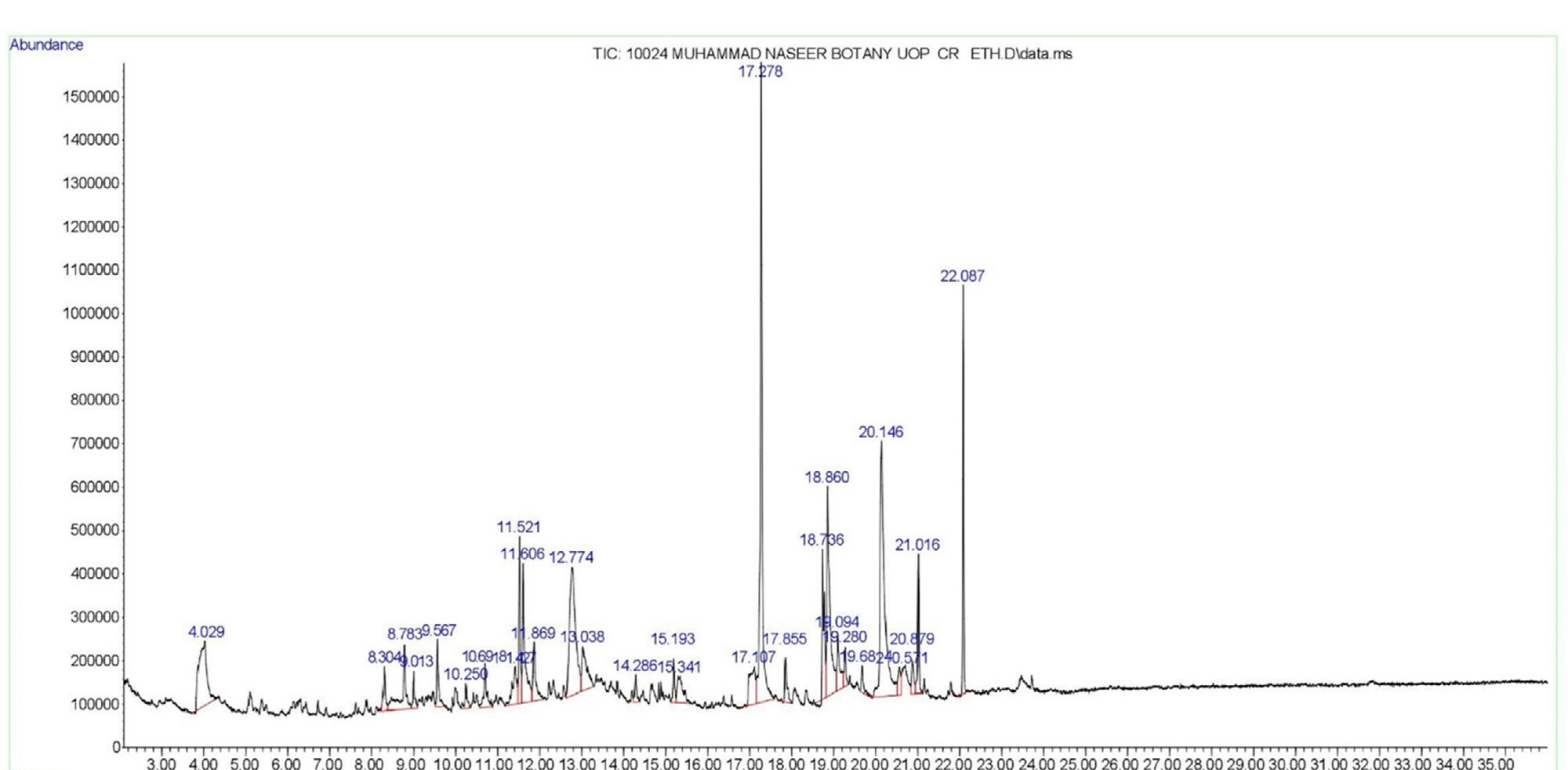
GC‐MS chromatogram of ethanolic extract of *Curio radicans*.

**Figure 2:**
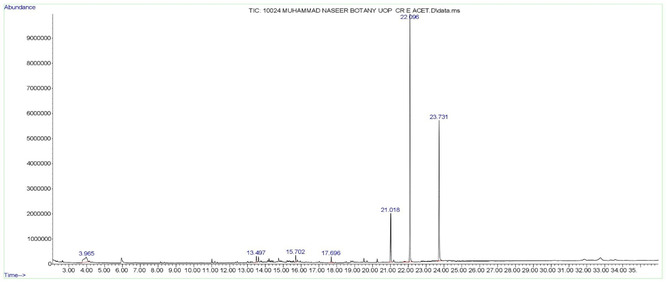
GC‐MS chromatogram of ethyl acetate extract of *Curio radicans*.

GC‐MS analysis revealed the probable phytochemicals present in both the ethanolic and ethyl acetate extracts of *Curio radicans*. A total of 29 phytoconstituents were identified in the ethanolic extract, while 7 phytoconstituents were found in the ethyl acetate extract (**Table** **1** and **2**; **Figure** **1** and **2**). The results showed that the most abundant compound in the ethanolic extract was 3,4‐Bis (Methoxycarbonyl)furan, accounting for 15.76%, followed by 4‐Imidazolidinone, 5‐(1‐methylpr…, at 14.17%. Other significant compounds included ethyl 1‐methylpipecolinate (9.84%), 9‐Octadecenoic acid (7.96%), glycerin (6.39%), phthalic acid, di (2‐propylpentyl… (5.38%)), and cyclopentanem ethanol, 3‐methylene‐(4.48%) (Table 1; Figure 1). In the ethyl acetate extract, Diisooctyl phthalate was found in the highest concentration (49.25%), followed by 1,3‐Benzenedicarboxylic acid (32.00%), hexanedioic acid, bis (2‐ethylhex… (8.85%), diglycerol (6.28%), 1‐Octadecene (1.35%), and behenic alcohol (1.28%) (Table 2; Figure 2).

**Table 1:** GC‐MS analysis of ethanolic extract of *Curio radicans*.

S. NO	Name/Compound	Formula	RT (Min)	Area (%)	Si	Molecular weight	Probability **%**
1	Glycerin	C_3_H_8_O_3_	4.029	6.39	2421	92.09	78
2	Benzene, 1‐methyl‐2‐nitro	C_7_H_7_NO_2_	8.3041	1.09	16 668	140.15	35
3	Acetonitrile, 2,2'‐iminobis‐	C_11_H_11_N_3_	8.783	3.11	2655	185.22	30
4	2,7‐Octadiene, 4‐methyl	C_9_H_16_	9.013	0.72	10 654	124.22	49
5	2‐Methoxy‐4‐vinylphenol	C_9_H_10_O_2_	9.567	1.55	24 419	150.17	94
6	3‐Tetradecyn‐1‐ol	C_14_H_26_O	10.250	0.63	69 492	210.36	27
7	2‐Methyl‐4‐(2,6,6‐trimethylcyclo…	C_15_H_24_O	10.698	1.38	67 872	220.35	38
8	Imidazo (1,2‐a) pyrimidine, 6‐meth.	C_6_H_5_N_3_	11.427	1.91	24 752	119.12	64
9	Sulfurous acid, dipentyl ester	C_17_H_36_O_3_S	11.521	2.81	78 606	320.5	35
10	Cyclopentane methanol, 3‐methylene‐	C_7_H_14_O	11.606	4.48	6601	114.1855	27
11	Propanal, 2‐propenylhydrazone	C_6_H_12_N_2_	11.869	1.87	6303	112.17	35
12	Ethyl 1‐methylpipecolinate	C_8_H_9_NO_2_	12.774	9.84	39 397	151.16	38
13	2‐Decenal, (E)‐	C_10_H_18_O	13.038	2.55	26 667	154.2493	38
14	Pyroquilon	C_11_H_11_NO	14.286	0.58	40 532	173.21	64
15	5,5,8a‐Trimethyl‐3,5,6,7,8,8a‐he	C_12_H_20_O	15.193	0.86	45 647	180.29	53
16	1‐(2,6‐Dimethyl‐phenyl)‐3‐piperi…	C_21_H_24_Cl_2_N_2_O	15.341	1.85	98 257	391.3	64
17	Quinoline‐4‐carbonitrile, 2‐methyl‐	C_10_H_6_N_2_	17.107	2.52	36 726	154.17	35
18	3,4‐Bis (Methoxycarbonyl)furan	C_10_H_11_BO_6_	17.278	15.76	49 177	238.00	32
19	Pyridine‐2,4‐diol, 3,5,6‐trimethyl	C_8_H_12_ClN	17.855	1.18	26 471	157.64	27
20	2,2‐Dimethyl‐4‐oxo‐1‐*p*‐tolyl‐cyc…	C_7_H_12_O_2_	18.736	3.78	83 646	128.17	30
21	9‐Octadecenoic acid	C_18_H_34_O_2_	18.860	7.96	129 353	282.5	99
22	*cis*‐Vaccenic acid	C_18_H_34_O_2_	19.094	2.03	129 339	282.5	94
23	8‐Quinolinol, 2‐(N‐tert‐butylfor…	C_13_H_15_N	19.280	0.87	84 419	185.26	35
24	2‐Methylsulfonyl‐5‐dimethylamino…	C_9_H_13_NO_2_S	19.684	0.71	84 767	199.27	38
25	4‐Imidazolidinone, 5‐(1‐methylpr…	C_7_H_12_N_2_OS	20.146	14.17	40 046	172.25	25
26	4‐Imidazolidinone, 5‐(2‐methylpr…	C_4_H_6_N_2_OS	20.571	0.94	40 045	130.168	25
27	Benzenemethanamine, 4‐methoxy	C_8_H_11_NO	20.879	0.98	16 769	137.1790	44
28	Hexanedioic acid, bis (2‐ethylhex	C_22_H_42_O_4_	21.016	2.09	196 965	370.6	95
29	Phthalic acid, di (2‐propylpentyl…	C_26_H_26_O_4_	22.087	5.38	207 709	402.5	91

**Table 2:** GC‐MS analysis of ethyl acetate extract of *Curio radicans*.

S. No	N/Compounds	Formula	RT (Min)	Area (%)	Si	Molecular weight	Probability %
1	Diglycerol	C_6_H_14_O_5_	3.965	6.28	35 351	166.17	74
2	Tetradecyl trifluoroacetate	C_16_H_29_F_3_O_2_	13.497	0.99	92 567	310.39	94
3	1‐Octadecene	C_18_H_36_	15.702	1.35	104 182	252.5	99
4	Behenic alcohol	C_22_H_44_O_2_	17.696	1.28	115 906	340.6	94
5	Hexanedioic acid, bis(2‐ethylhex)	C_22_H_42_O_4_	21.018	8.85	196 971	370.6	99
6	Diisooctyl phthalate	C_24_H_38_O_4_	22.096	49.25	207 658	390.6	91
7	1,3‐Benzenedicarboxylic acid	C_8_H_4_Na_2_O_4_	23.731	32.00	207 811	210.09	94

### 3.2 Genoprotective Effects of the Ethanolic Extract of *Curio Radicans*

**Figure 3:**
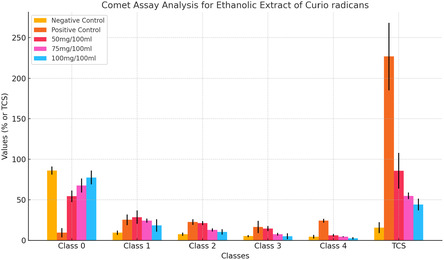
Commet assay of genomic DNA of human lymphocytes exposed to ethanolic extract of *Curio radicans* for 24 h.

The genoprotective effects of the ethanolic extract of *Curio radicans* were evaluated using the comet assay on human lymphocyte DNA after 24 h of exposure. The findings revealed that the highest degree of DNA damage was observed in lymphocytes treated with the positive control, hydrogen peroxide (H_2_O_2_), with a total comet score (TCS) of 226.7 ± 41.6. When lymphocytes were treated with the ethanolic extract of *C. radicans* at varying concentrations (50 mg/100 mL, 75 mg/100 mL, and 100 mg/100 mL), a dose‐dependent reduction in DNA damage was observed. At 50 mg/100 mL, the TCS was 85.7 ± 22.0, which further decreased to 54.7 ± 4.0 and 44.0 ± 7.2 at 75 mg/100 mL and 100 mg/100 mL, respectively. These reductions in TCS were statistically significant (*p* < 0.001) compared to the positive control. These results demonstrate the genoprotective potential of the ethanolic extract of *C. radicans*, with a significant reduction in DNA damage across all tested concentrations, as shown in **Table** **3**; **Figure** **3**.

**Table 3:** Comet assay of genomic DNA of human lymphocytes exposed to ethanolic extract of *Curio radicans* for 24 h.

Class	Negative control (only lymphocytes)	Positive control (lymphocytes + H_2_O_2_)	50 mg/100 mL	75 mg/100 mL	100 mg/100 mL
**Class 0**	86.0 ± 5.0	9.5 ± 5.7	54.3 ± 7.1	67.5 ± 8.6	77.4 ± 8.5
**Class 1**	9.5 ± 2.6	25.4 ± 6.6	28.5 ± 8.1	24.3 ± 2.5	18.5 ± 7.7
**Class 2**	7.6 ± 2.0	22.6 ± 3.4	21.4 ± 2.5	13.0 ± 2.0	10.5 ± 3.4
**Class 3**	5.3 ± 1.3	16.5 ± 7.6	14.6 ± 3.2	7.6 ± 1.7	5.0 ± 3.6
**Class 4**	4.5 ± 2.2	24.3 ± 2.4	6.3 ± 1.5	4.5 ± 0.6	2.6 ± 1.4
**TCS**	15.7 ± 6.6	226.7 ± 41.6	85.7 ± 22.0*	54.7 ± 4.0*	44.0 ± 7.2*

### 3.3 Genoprotective Effects of the Ethyl Acetate Extract of *Curio Radicans*

**Figure 4:**
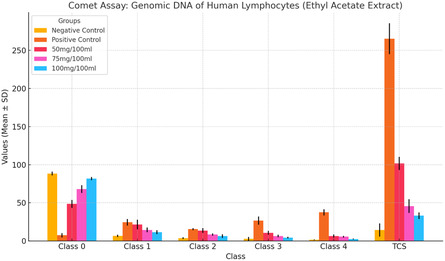
Commet assay of genomic DNA of human lymphocytes exposed to ethyl acetate extract of *Curio radicans* for 24 h.

The genoprotective effects of the ethyl acetate extract of *Curio radicans* were evaluated using the comet assay on human lymphocyte DNA after 24 h of exposure. The results revealed the highest degree of DNA damage in lymphocytes treated with the positive control, hydrogen peroxide (H_2_O_2_), with a TCS of 265.3 ± 20.4. When lymphocytes were treated with varying concentrations of the ethyl acetate extract of *C. radicans* (50 mg/100 mL, 75 mg/100 mL, and 100 mg/100 mL), a dose‐dependent reduction in DNA damage was observed. At 50 mg/100 mL, the TCS was 101.7 ± 8.6, which decreased to 45.7 ± 9.0 at 75 mg/100 mL, and further to 33.0 ± 4.3 at 100 mg/100 mL. These reductions were statistically significant (*p* < 0.0001) when compared to the positive control. These results demonstrate the genoprotective potential of the ethyl acetate extract of *C. radicans*, with significant reductions in DNA damage observed at all tested concentrations, as shown in **Table** **4**; **Figure** **4**.

**Table 4:** Comet assay of genomic DNA of human lymphocytes exposed to ethyl acetate extract of *Curio radicans* for 24 h.

Class	Negative control (only lymphocytes)	Positive control (lymphocytes + H_2_O_2_)	50 mg/100 mL	75 mg/100 mL	100 mg/100 mL
**Class 0**	88.4 ± 2.5	7.4 ± 2.8	48.5 ± 5.1	68.0 ± 5.0	81.6 ± 2.1
**Class 1**	6.6 ± 1.5	24.4 ± 4.3	21.6 ± 6.3	14.5 ± 3.0	11.5 ± 2.5
**Class 2**	3.5 ± 1.2	15.5 ± 1.3	13.5 ± 3.1	8.5 ± 1.5	6.4 ± 2.4
**Class 3**	2.6 ± 2.5	26.6 ± 5.3	10.6 ± 2.6	6.4 ± 1.7	4.3 ± 1.0
**Class 4**	1.4 ± 0.48	37.5 ± 4.0	6.4 ± 2.1	5.6 ± 1.1	2.4 ± 0.56
**TCS**	14.3 ± 8.6	265.3 ± 20.4	101.7 ± 8.6*	45.7 ± 9.0**	33.0 ± 4.3**

### 3.4 Antioxidant Activity

**Figure 5:**
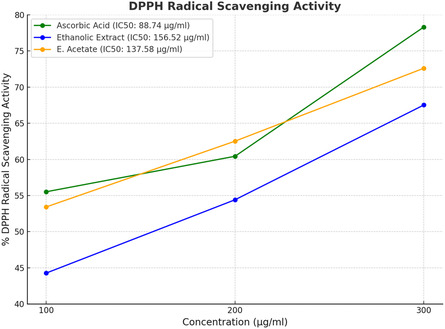
Antioxidant activity of *Curio radicans* of ethanolic and ethyl acetate extracts.

The antioxidant activity of ethanolic and ethyl acetate extracts of *Curio radicans* is presented in **Table** **5**; **Figure** **5**. The results revealed that ascorbic acid demonstrated the highest antioxidant activity, with a % DPPH radical scavenging activity of 78.31 ± 1.23 at 300 μg/ml. The ethyl acetate extract exhibited moderate activity (72.61 ± 1.65 at 300 μg/ml), followed by the ethanolic extract, which showed lower antioxidant activity (67.52 ± 0.44 at the same concentration). The antioxidant activity of all samples increased in a dose‐dependent manner. The standard (ascorbic acid) exhibited significantly higher DPPH radical scavenging activity than both the ethanolic and ethyl acetate extracts. The lowest antioxidant activity (44.27 ± 1.32) was observed for the ethanolic extract at 100 μg/ml. The concentrations of the studied samples required to scavenge 50% of the DPPH radicals (IC_50_) were also determined in this study. The IC_50_ values for the ethanolic and ethyl acetate extracts were 156.52 μg/ml and 137.58 μg/ml, respectively, while the IC_50_ value of ascorbic acid was 88.74 μg/ml, indicating its superior antioxidant potential.

**Table 5:** Antioxidant activity of ethanolic and E. acetate extracts of *Curio radicans*.

Sample	Conc. (μg/ml)	% DPPH radical scavenging activity	IC_50_ (μg/ml)
Ascorbic acid	100	55.52 ± 0.37	88.74
	200	60.43 ± 0.63	
	300	78.31 ± 1.23	
Ethanolic extract	100	44.27 ± 1.32	156.52
	200	54.41 ± 0.65	
	300	67.52 ± 0.44	
E. acetate	100	53.42 ± 0.53	137.58
	200	62.52 ± 1.42	
	300	72.61 ± 1.65	

## 4 Discussion

The use of GC‐MS for chemo‐profiling plant extracts plays a crucial role in fields such as pharmacognosy and pharmaceutical biotechnology. Recent studies emphasize the significance of integrating GC with MS to analyze complex mixtures, including essential oils, hydrocarbons, and secondary metabolites.^[^ ^13^ ^]^ This technique enables the efficient separation, identification, and detection of individual components within these mixtures.^[^ ^14^ ^]^ The ethanolic and ethyl acetate extracts of *Curio radicans* revealed the presence of several bioactive compounds with distinct retention times (min), as summarized in Table 1. The GC‐MS analysis of the ethanolic extract identified 29 compounds, including therapeutically significant ones such as phthalic acid, di(2‐propylpentyl), hexanedioic acid, bis(2‐ethylhexyl), quinoline‐4‐carbonitrile, 2‐methyl, and benzenemethanamine, 4‐methoxy. In contrast, the ethyl acetate extract revealed seven compounds (Table 2), with key therapeutic constituents including behenic alcohol, 1,3‐benzenedicarboxylic acid, and diisooctyl phthalate. These findings align with the study by Ezez et al.^[^ ^15^ ^]^ which reported similar phytochemical constituents in the leaf extract of *Withania somnifera*. Among the compounds identified in the ethanolic and ethyl acetate fractions, some exhibit genoprotective properties. For instance, methylbenzaldehyde‐N‐allyl, a benzaldehyde derivative, demonstrates genoprotective effects primarily by modulating cellular antioxidant pathways and reducing oxidative stress‐induced DNA damage.^[^ ^16^ ^]^ By scavenging ROS and enhancing the activity of antioxidant enzymes such as superoxide dismutase and catalase, methylbenzaldehyde‐N‐allyl plays a crucial role in minimizing DNA strand breaks and mutations. These findings are consistent with ref. [17]. Phthalates have been shown to induce single‐ and double‐strand DNA breaks. Studies suggest that they are potential genotoxic agents in human lymphocytes and animal tissues.^[^ ^18^ ^]^ Quinoline derivatives exhibit antioxidant or protective properties due to their ability to scavenge free radicals.^[^ ^19^ ^]^ The methyl group at position 2 may reduce reactivity, which could alter genotoxic potential.^[^ ^20^ ^]^ n‐Hexadecanoic acid has been reported to possess antioxidant, pesticide, lubricant, and antiandrogenic properties.^[^ ^21^ ^]^ These findings align with those of ref. [22] who identified similar phytochemicals during the GC‐MS analysis of *Petiveria alliaceae* L. Hexanedioic acid, bis(2‐ethylhexyl), is recognized for its potent antimicrobial activity, while n‐hexadecanoic acid also demonstrates significant anti‐inflammatory and antioxidant effects. Behenic alcohol is widely used in medicine as an antiseptic, disinfectant, and antidote. Additionally, *cis*‐vaccenic acid has therapeutic relevance in managing sickle cell anemia and beta‐thalassemia.^[^ ^23^ ^]^ Phthalic acid, di(2‐propylpentyl), serves as a plasticizer in products such as nail polishes and hair sprays and is also used as a solvent and perfume fixative. Furthermore, 9‐octadecenoic acid exhibits notable anticancer,^[^ ^24^ ^]^ anti‐inflammatory,^[^ ^25^ ^]^ and antiandrogenic properties.^[^ ^26^ ^]^ Benzenemethanamine, 4‐methoxy (also referred to as phthalic acid), has been shown to inhibit microbial growth and disrupt cell proliferation.^[^ ^27^ ^]^ Genotoxic studies are conducted to assess potential damage resulting from prolonged use of plant extracts. These studies are essential for identifying the toxic effects of various drugs and ensuring their safety.^[^ ^28^, ^29^, ^30^, ^31^ ^]^ Phthalates have been shown to cause single‐ and double‐strand DNA breaks, indicating their potential genotoxicity in human lymphocytes and animal tissues.^[^ ^32^, ^33^, ^34^ ^]^ In this study, the ethanolic extract of *C. radicans* showed genoprotective effects in human lymphocytes using the comet assay. Hydrogen peroxide (H_2_O_2_) caused the highest DNA damage (TCS: 226.7 ± 41.6). Treatment with the extract reduced damage in a dose‐dependent manner: 50 mg/100 mL (TCS: 85.7 ± 22.0), 75 mg/100 mL (TCS: 54.7 ± 4.0), and 100 mg/100 mL (TCS: 44.0 ± 7.2), all significant (*p* < 0.001) compared to the control. These results demonstrate the genoprotective potential of the ethanolic extract of *C. radicans*, with a significant reduction in DNA damage across all tested concentrations, as shown in Table 3. While the ethyl acetate extract of *C. radicans* showed genoprotective effects in human lymphocytes using the comet assay. H_2_O_2_ caused the highest DNA damage (TCS: 265.3 ± 20.4). The extract reduced damage dose‐dependently: 50 mg/100 mL (TCS: 101.7 ± 8.6), 75 mg/100 mL (TCS: 45.7 ± 9.0), and 100 mg/100 mL (TCS: 33.0 ± 4.3), all significant (*p* < 0.0001) compared to the control. These results demonstrate the genoprotective potential of the ethyl acetate extract of *C. radicans*, with significant reductions in DNA damage observed at all tested concentrations, as shown in Table 4. The antioxidant activity of *Curio radicans* extracts was evaluated using DPPH assay. Ascorbic acid showed the highest activity (78.31 ± 1.23% at 300 μg/ml, IC_50_: 88.74 μg/ml). The ethyl acetate extract exhibited moderate activity (72.61 ± 1.65%, IC_50_: 137.58 μg/ml), while the ethanolic extract had the lowest activity (67.52 ± 0.44%, IC_50_: 156.52 μg/ml). Activity increased dose‐dependently, with the ethanolic extract showing only 44.27 ± 1.32% at 100 μg/ml (Table 5). These results are in line with ref. [8] who reported the similar results with genotoxic and antioxidant potential of *Achillea millefolium* and *Chaerophyllum villosum.*

## 5 Conclusion

The study underscores the promising therapeutic potential of *Curio radicans* extracts, particularly the ethyl acetate fraction, which exhibited remarkable genoprotective and moderate antioxidant properties. The identification of key bioactive compounds through GC‐MS provides valuable insight into the chemical composition of these extracts, which could be leveraged for future pharmacological applications. The significant dose‐dependent reduction in DNA damage and the observed antioxidant activity suggest that *C. radicans* may be a valuable source of natural compounds with therapeutic efficacy. Further investigation into the isolation and mechanistic understanding of these active constituents will be essential for the development of novel, natural‐based interventions for disease prevention and treatment. The findings advocate for the potential use of *C. radicans* as a functional therapeutic agent in modern medicine.

## Conflict of Interest

The authors declare no conflict of interest.

## Author Contributions

**Muhammad Naseer**: writing—original draft preparation, conceptualization, resources, validation and data curation; **Muhammad Adil**: supervision, project administration; **Mikhlid H. Almutairi** and **Sarir Ahmad**: writing—review & editing, formal analysis and provided funding; **Sarir Ahmad** and **Abdulwahed Fahad Alrefaei**: visualization.

## Ethical Approval and Consent to Participate

Ethical permission was not needed from the institutional committee for this study, and the informed consent was obtained from the participant.
